# Supplementary material for: Strict molecular sieving over electrodeposited 2D-interspacing-narrowed graphene oxide membranes
Source: Nat Commun. 2017 Oct 10;8:825. doi: 10.1038/s41467-017-00990-x (PMC5635034; doi:10.1038/s41467-017-00990-x)
Supplement: Supplementary file 1 — Supplementary Information [file 41467_2017_990_MOESM1_ESM.pdf]

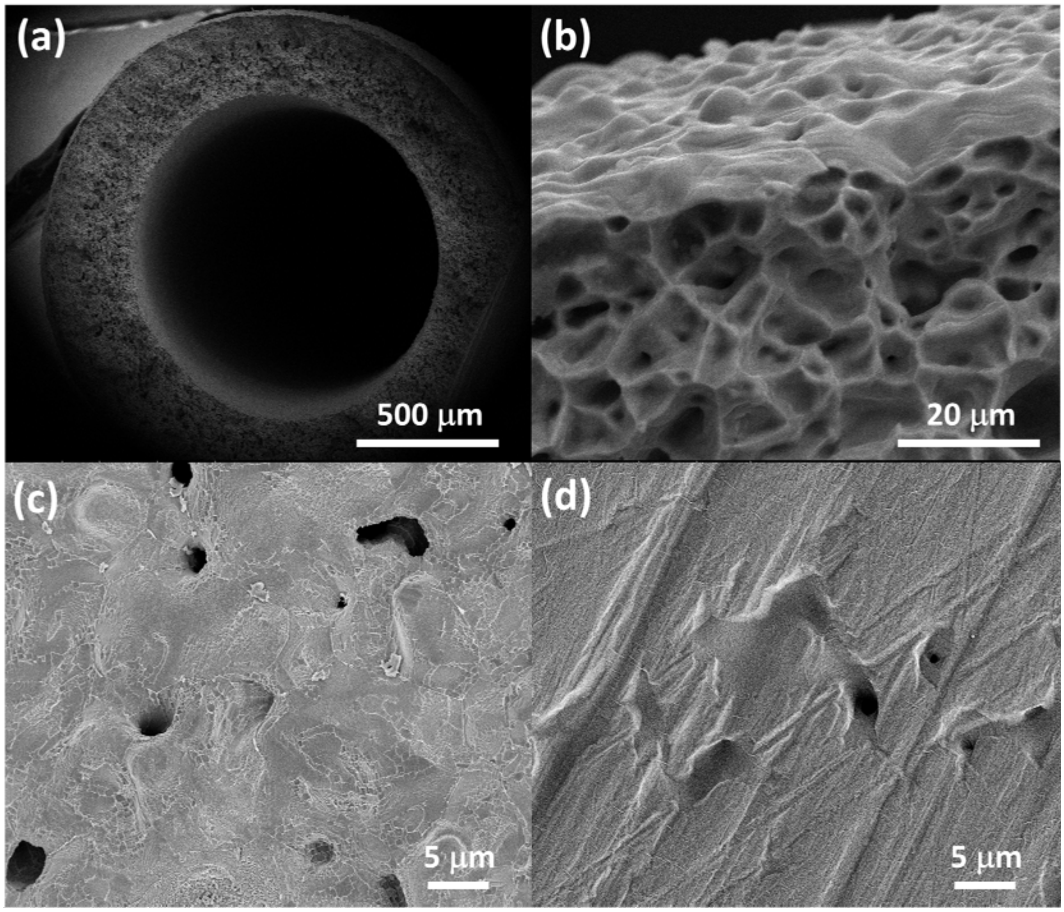


**Supplementary Figure 1**. **Morphology of porous stainless steel hollow fibres.** (**a**) & (**b**) cross-section and (**c**) surface SEM views of as prepared PSSHFs; (**d**) surface SEM image of PSSHF after the step-by-step polish treatments.

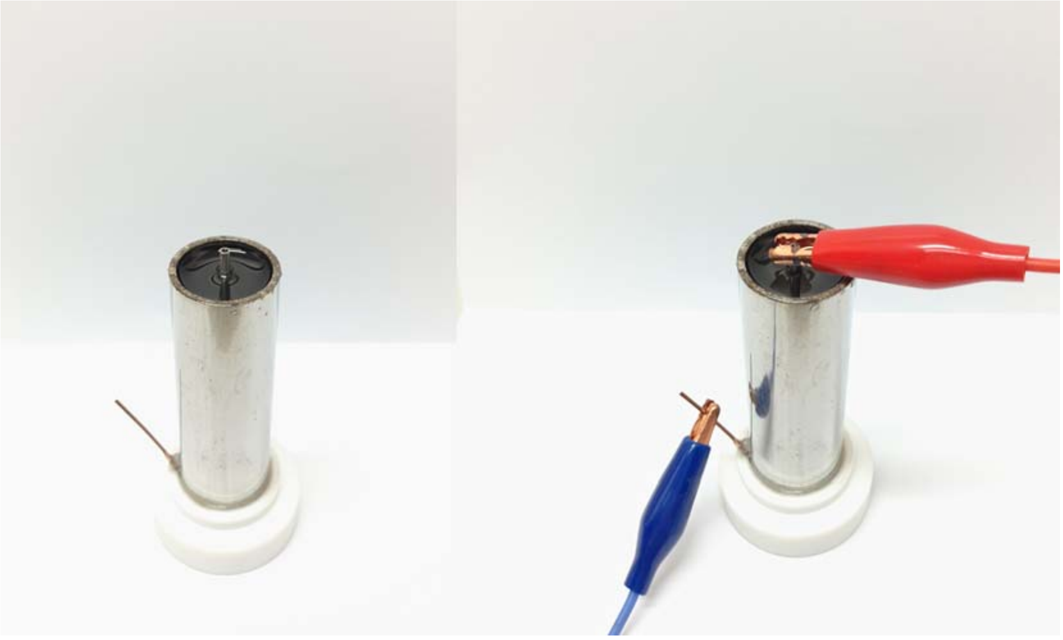

**Supplementary Figure 2. Pictures of the electrolytic cell for GO electrophoresis deposition.**

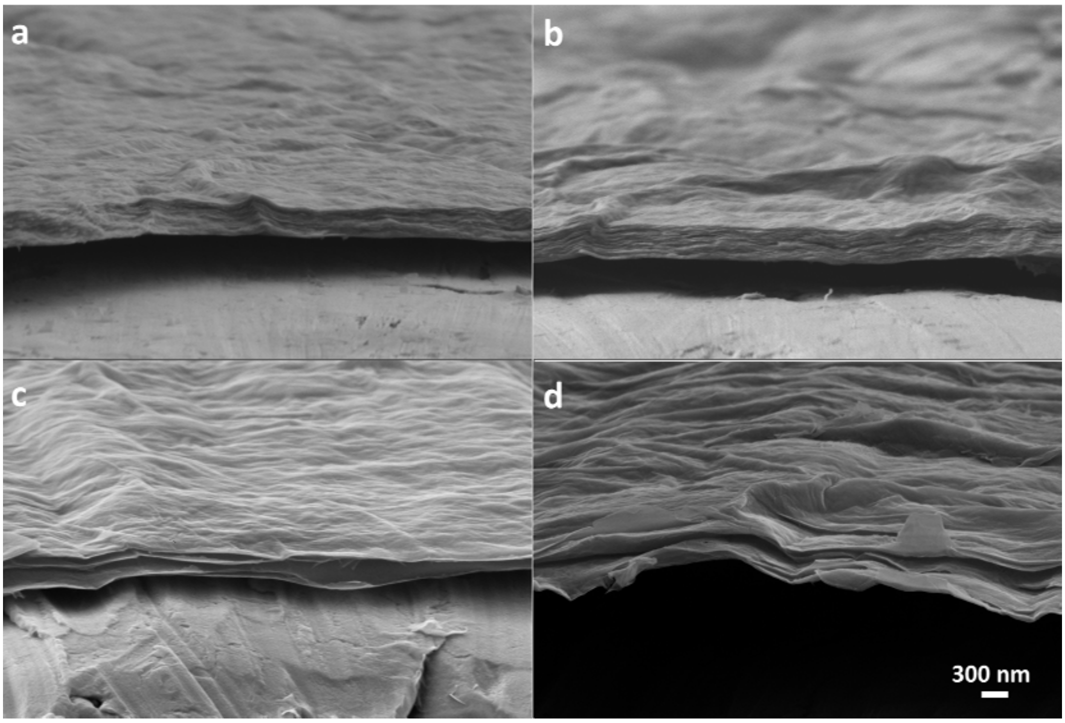


**Supplementary Figure 3 Effects of DC voltage on the morphology of ED-GO layer.** (**a**) 4.5 V; (**b**) 6 V; (**c**) 9 V and (**d**) 12 V. For the membrane prepared under 4.5 and 6 V, the GO flakes are well stacked. In contrast, the membranes prepared by higher voltages show hollow structure and larger wrinkles. It may be caused by bubbles released from the electrode.

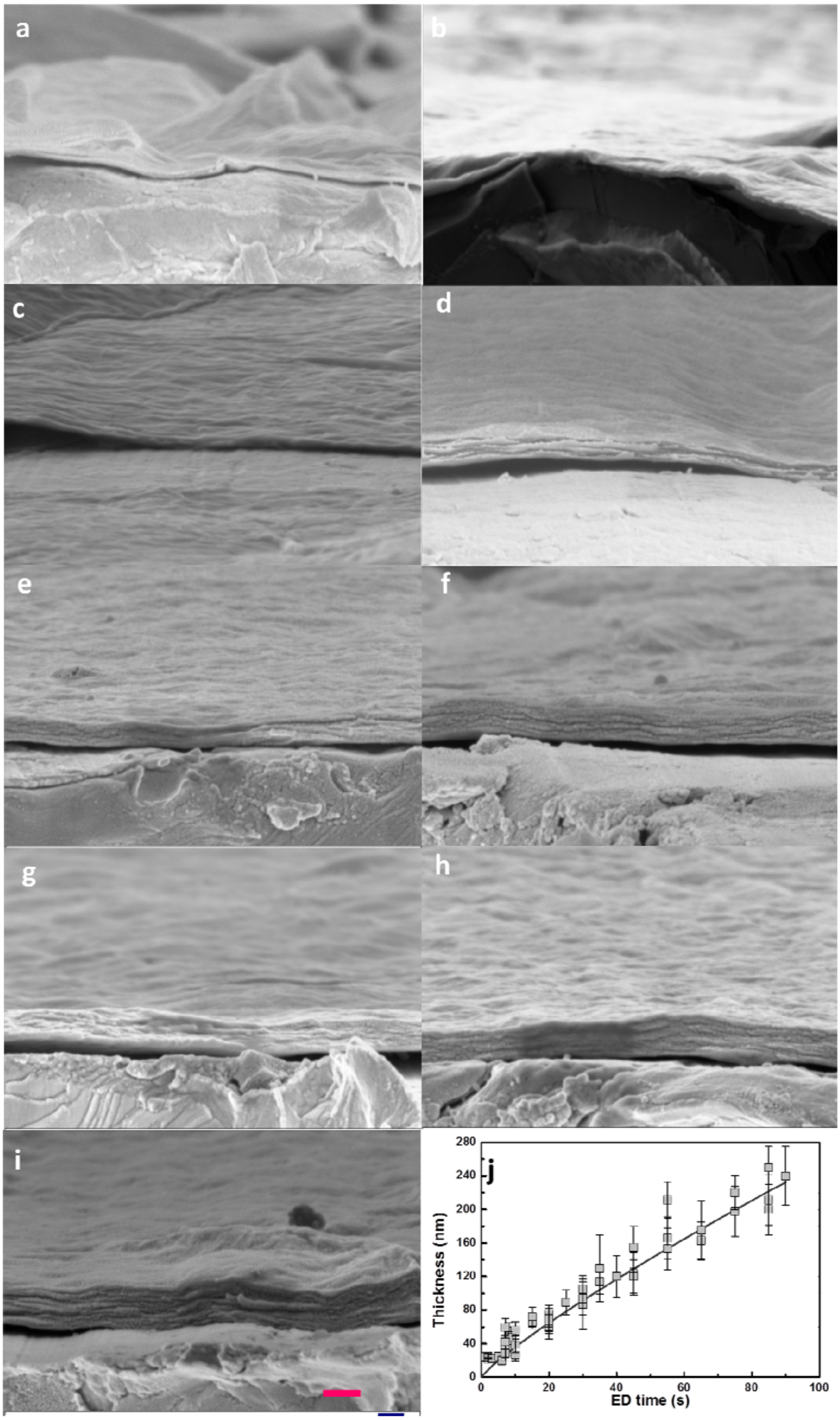


**Supplementary Figure 4. Effects of ED time on the morphology of ED-GO@PSSHF membranes.** (**a-i**) Cross-section views of ED-GO layer after 5-85 s ED with 10 s interval (Scale bar 200 nm) and (**j**) GO layer thickness variation with ED time. ($V_{WE}$-$V_{CE}$ =4.5V and 9 mm electrode spacing)

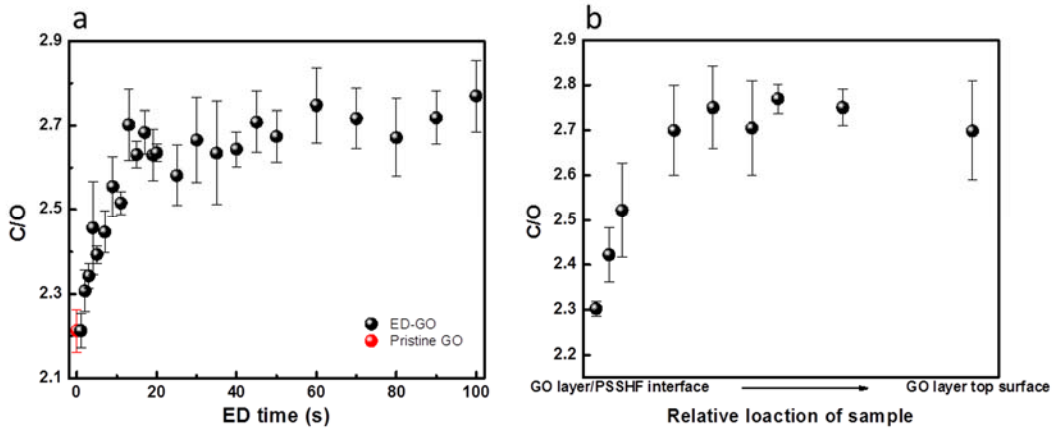


**Supplementary Figure 5**. **Effects of electro-deposition time on the composition of ED-GO@PSSHF membranes.** (**a**) C/O of the ED-GO layers at different time ($V_{WE}$ - $V_{CE}$ =4.5V and 9 mm electrode spacing) and (**b**) C/O depth profile from membrane/support interface to top surface of an ED-GO layer ($V_{WE}$ - $V_{CE}$ =4.5V, 9 mm electrode spacing, 80 s). For the C/O depth profile, the samples were prepared by using copper adhesive tape to exfoliate the ED-GO@PSSHF membrane from the top surface to the interface step-by-step. Therefore, the results in Supplementary Figure 5b only show the relative location within the membrane. It’s noted that we also employed in-site Ar ion sputter in XPS chamber to etch the membrane with a reliable thickness interval. But the results indicate that GO sample was deoxygenated significantly due to the Ar ion thermal effect or other interactions during sputtering.

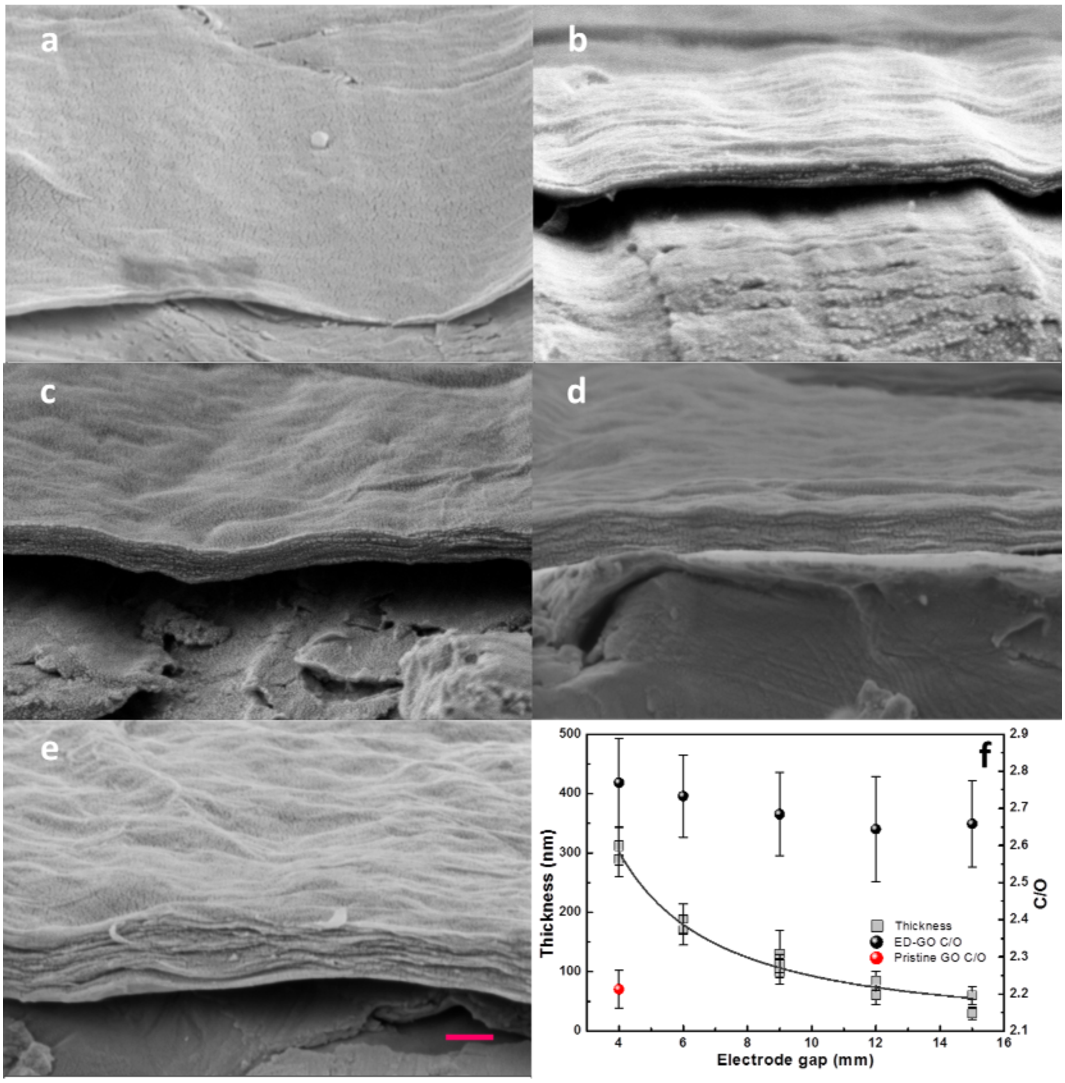


**Supplementary Figure 6**. **Effects of the electrode spacing on the morphology of ED-GO@PSSHF membranes.** Cross-section views of GO layer with different electrode spacing (**a**) 15, (**b**) 12, (**c**) 9, (**d**) 6 and (**e**) 4 mm (scale bar 200 nm); (**f**) Effects of electrode spacing on the thickness and C/O of ED GO layer ($V_{WE}$ - $V_{CE}$ =4.5V, 1mg/mL GO, 35 s).

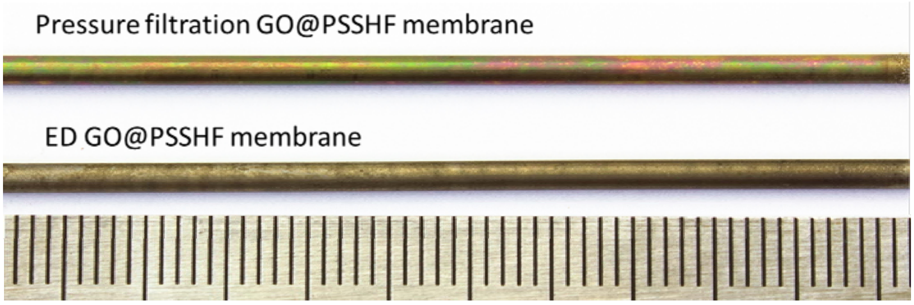


**Supplementary Figure 7. Optical image of PSSHF supported GO membranes**. The GO thin membrane normally is brown since the functional groups of GO reflect the corresponding light colour, while reduced GO and graphene membrane is black owing to the weak light reflection. Herein, the colour of ED GO@PSSHF membrane is slightly darker than that of filtration membrane, empirically indicating that the former membrane was mildly reduced during the deposition.

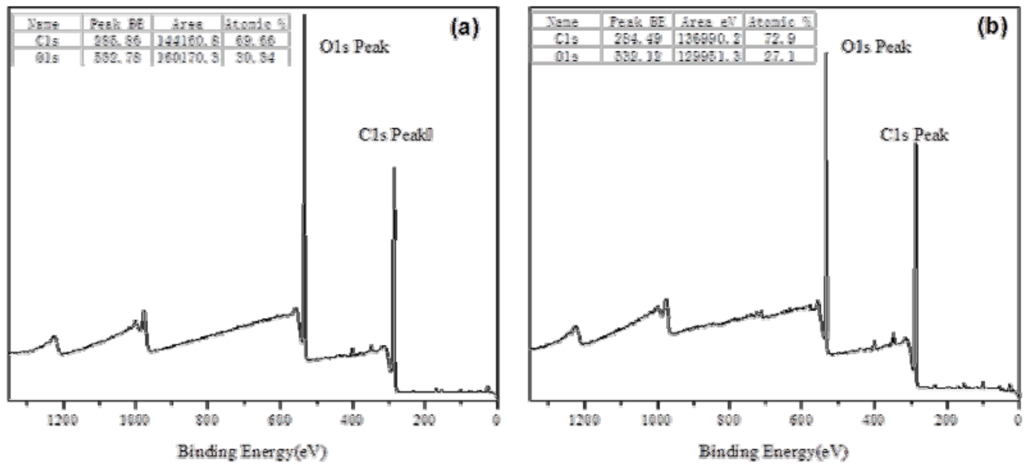


**Supplementary Figure 8. XPS measurements of pristine and ED-GO samples.**

Survey of pristine GO (**a**) and ED-GO (**b**).

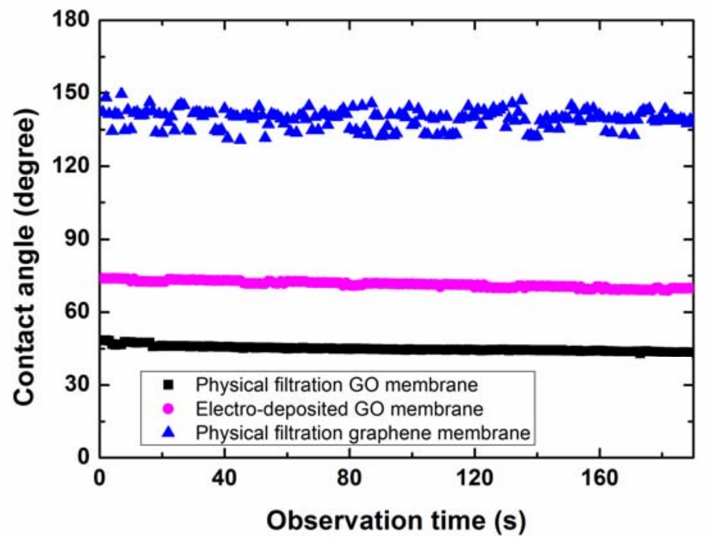


**Supplementary Figure 9**. **Dynamic water contact angle of water drop on the surface of ED-GO, filtration prepared GO and filtration prepared graphene membranes.** The interactions between water droplets and the membrane surface were further investigated by dynamic WCA. Unlike the graphene membrane, which displayed a dynamic WCA with fluctuation between 135$^o$ and 149$^o$, the WCA of pristine GO and ED-GO membranes decreased with time from 48.4$^o$ to 43.2$^o$ and 73.9$^o$ to 69.6$^o$, respectively, indicating that water can slightly access the bulk of GO membranes.

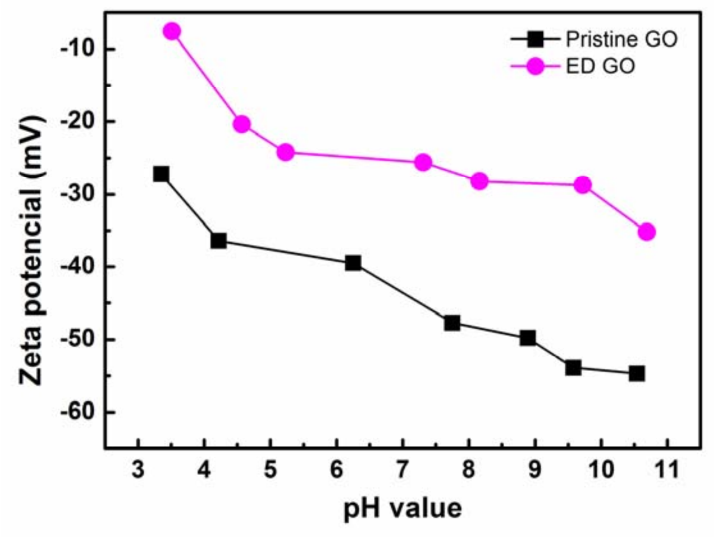


**Supplementary Figure 10. Zeta potential of pristine GO and ED-GO samples in the pH range 3-11.**

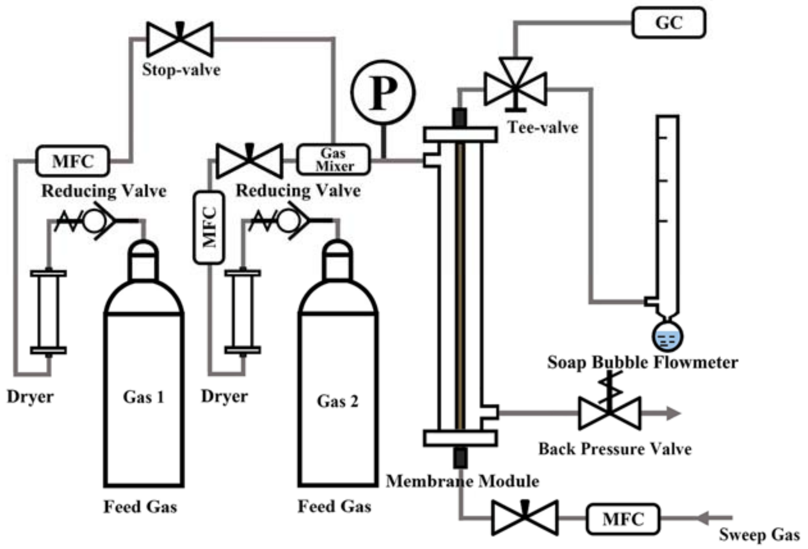


**Supplementary Figure 11**. **Schematic of the membrane system for gas permeance**

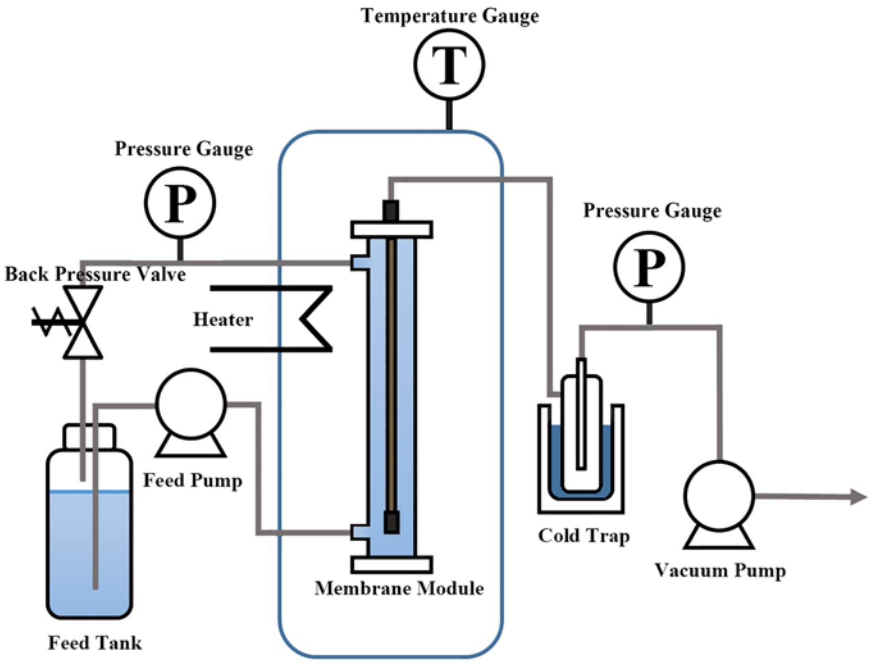


**Supplementary Figure 12**. **Schematic of the membrane system for the liquid separation**

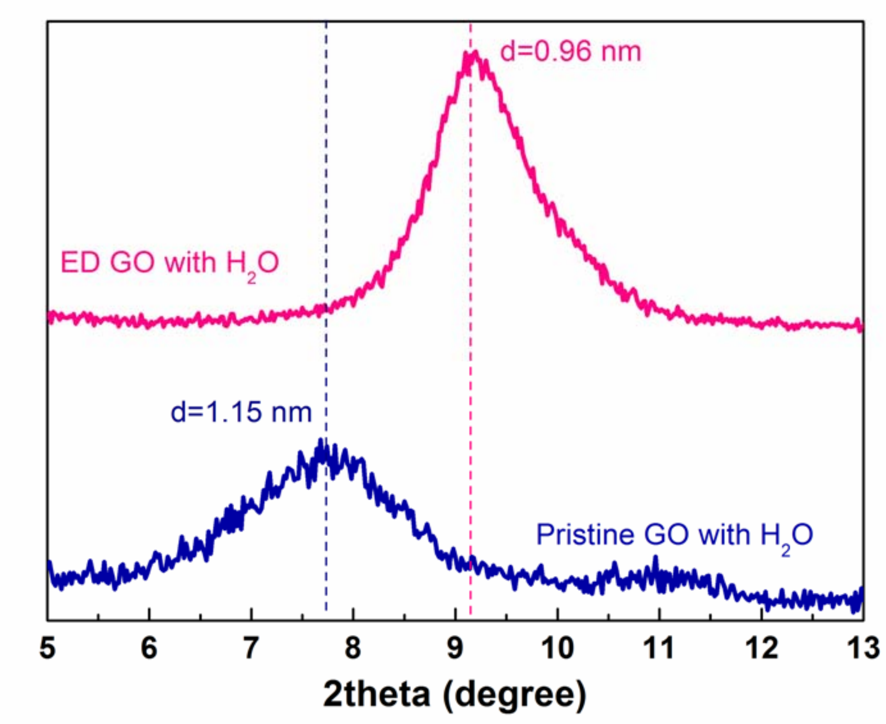


**Supplementary Figure 13 XRD patterns of pristine GO and ED-GO samples treated with water drops.**

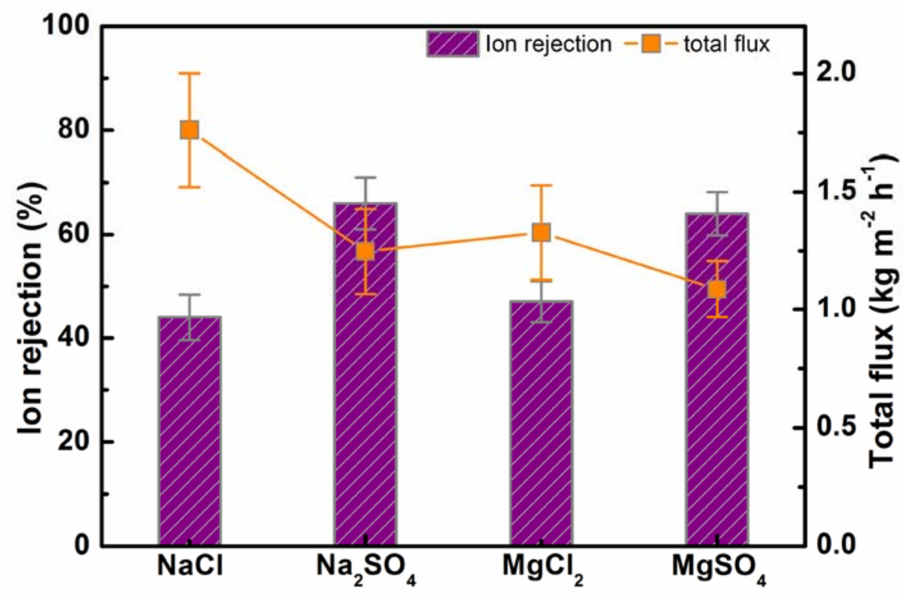


**Supplementary Figure 14 The saline water separation performance over pristine GO@PSSHF membrane with VMD method** (feed concentration 0.1 mol $L^{-1}$, 60 $^{o}C$, the membrane was prepared by pressure-assisted filtration)**.**

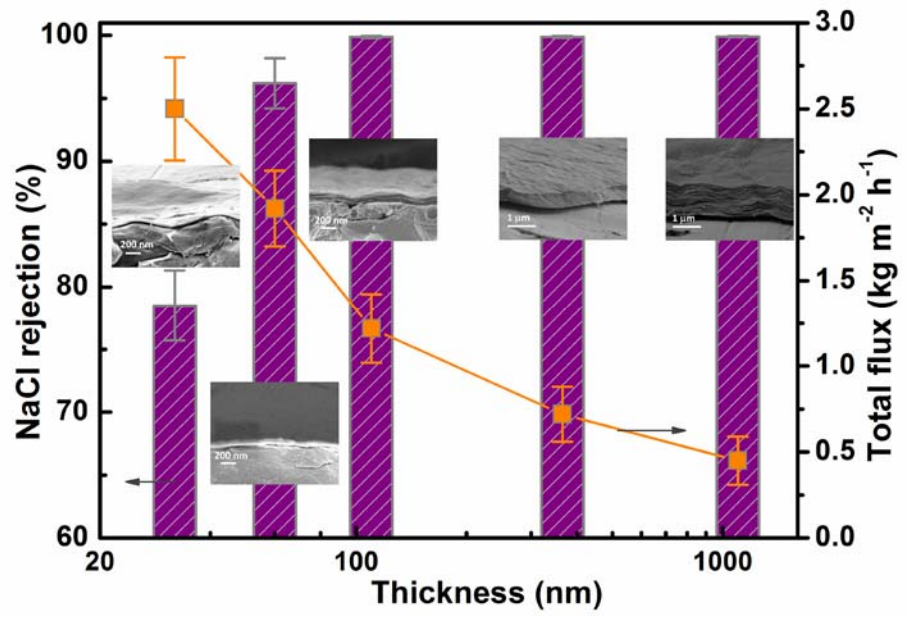


**Supplementary Figure 15 Effects of membrane thickness on the desalination.** The separation performance of NaCl solution (0.1 mol $L^{-1}$) over ED-GO@PSSHF membranes with different thickness through pervaporation method at 60 $^{o}$C (Membranes were prepared under the conditions of 4.5 V and 9 mm electrode space with different time).

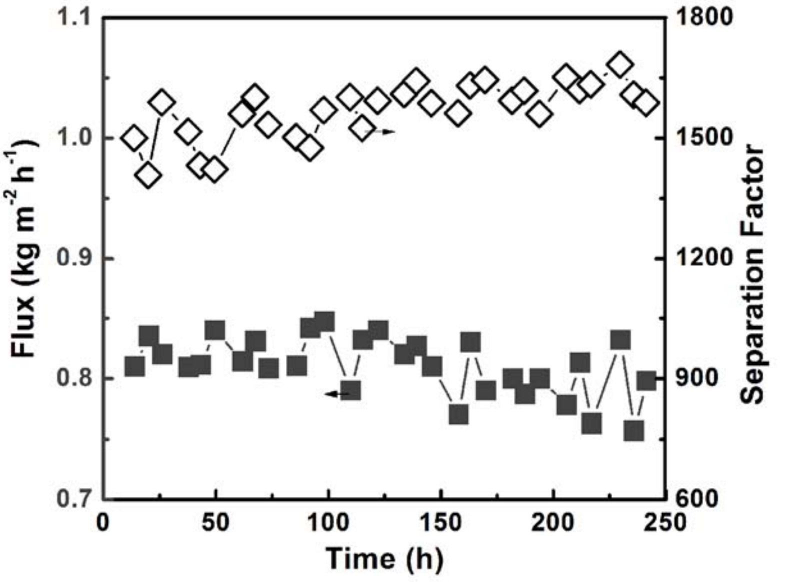


**Supplementary Figure 16 The long-term stability of ED-GO@PSSHF membrane in the pervaporation of ethanol-$H_2O$ binary mixture (1:1) at 70 $^oC$.**

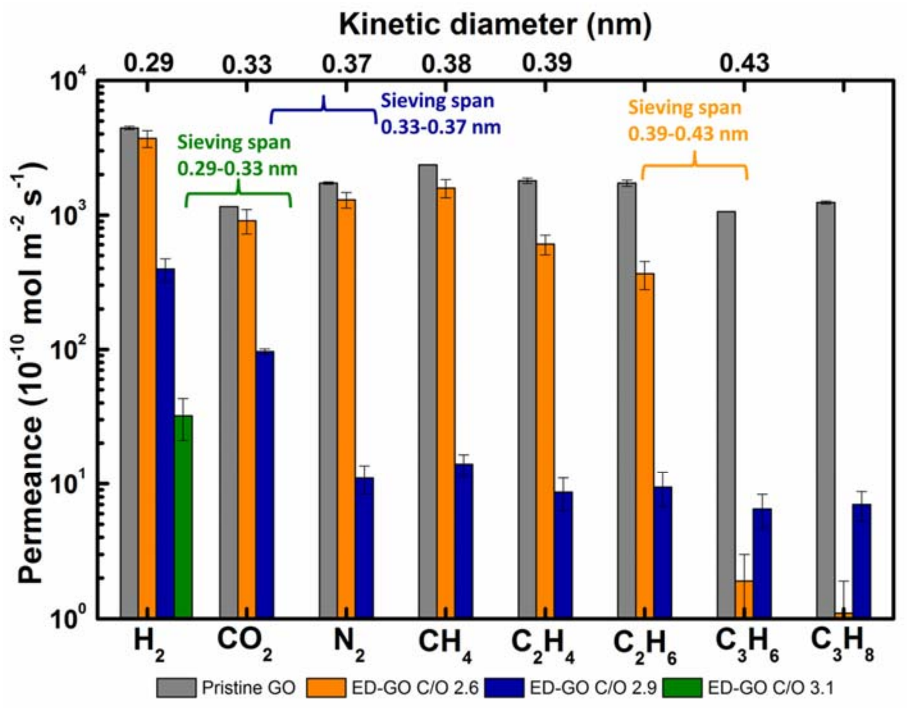


**Supplementary Figure 17 Single gas permeance of pristine GO@PSSHF membrane and ED-GO@PSSHF membranes with different reduction.** The pristine GO@PSSHF membrane was prepared by pressure assisted filtration. The ED-GO@PSSHF membranes with orange, blue and green symbol were prepared under 4.5 V, 9 V and 12 V. With higher voltage, the deposited GO layer was further reduced, in which the C/O of membrane was increased from ~2.2 of pristine GO to ~2.6, 2.9 and 3.1 of ED-GO membranes. Correspondingly, the sieving point was down-shifted to the smaller scale.The sieving span was shift from > 0.43 nm for pristine GO membrane to 0.41±0.02, 0.35 ±0.02 and 0.31±0.02 nm of the three ED-GO@PSSHF membranes. On the other hand, deeply reduced ED-GO membranes exhibit remarkably enhanced mass diffusion resistance. In this way, the membrane with green symbol is nearly gas tight because that the permeance of $H_2$ was reduced by two orders of magnitude and the larger gases were undetectable.

**Supplementary Table 1** The composition of functional groups in pristine and ED-GO measured by XPS.

| | Pristine GO | | Electrodeposited GO* | |
|---|---|---|---|---|
| | Position (eV) | Atomic % | Position (eV) | Atomic % |
| C-C | 284.4 | 30.1 | 284.4 | 33.0 |
| C-OH | 285.2 | 24.5 | 285.0 | 29.1 |
| C-O-C | 286.6 | 12.9 | 286.3 | 6.5 |
| C=O | 287.4 | 20.3 | 287.0 | 25.0 |
| O=C-OH | 288.7 | 12.2 | 288.7 | 6.4 |

* 1 mg $mL^{-1}$ GO suspension, 9 mm electrode spacing, 35 s

**Supplementary Table 2** The desalination of NaCl and $Na_2SO_4$ solution over ED-GO@PSSHF membrane with VMD method (0.1 mol $L^{-1}$, 60 $^oC$).

| No | NaCl solution | | | $Na_2SO_4$ solution | | |
|---|---|---|---|---|---|---|
| | $R_{Na+}$ (%) | $R_{Cl-}$ (%) | Flux (kg $m^{-2}$ $h^{-1}$) | $R_{Na+}$ (%) | $R_{SO42-}$ (%) | Flux (kg $m^{-2}$ $h^{-1}$) |
| 1 | 99.88 | 99.85 | 1.10 | 99.98 | 99.97 | 0.75 |
| 2 | 99.83 | 99.85 | 1.32 | 99.96 | 99.98 | 0.63 |
| 3 | 99.87 | 99.84 | 1.24 | 99.99 | 100 | 0.84 |
| Av. | 99.86±0.10 | 99.85±0.07 | 1.22±0.09 | 99.98±0.01 | 99.98±0.01 | 0.74±0.09 |
