# Supplementary material for: Strict molecular sieving over electrodeposited 2D-interspacing-narrowed graphene oxide membranes
Source: Nat Commun. 2017 Oct 10;8:825. doi: 10.1038/s41467-017-00990-x (PMC5635034; doi:10.1038/s41467-017-00990-x)
Supplement: Supplementary file 3 — Description of Additional Supplementary Files [file 41467_2017_990_MOESM3_ESM.pdf]

Description of Additional Supplementary Files

File Name: Supplementary Data 1
Description: Current, i.e. I (mA), variation with fixed DC voltages of V WE -V CE in the GO electrophoresis deposition. The V WE -V CE was fixed on 1.5, 1.8, 2.1, 2.4, 2.7, 3.0, 3.3, 3.6, 3.9, 4.2, 4.5, 4.8 and 5.1 V, respectively.

File Name: Supplementary Data 2
Description: Cyclic voltammetry (CV) results (5 th , 15 th , 25 th and 35 th cycle) of GO sample on the glass-carbon electrode at room temperature.

File Name: Supplementary Data 3
Description: Time dependence of voltages V WE -V CE and V CE in the three-electrode system for GO deposition with a V WE of 3.2 V

File Name: Supplementary Data 4
Description: X-ray diffraction (XRD) results of pristine GO and ED-GO (2 theta=5-30 o)

File Name: Supplementary Data 5
Description: Atomic force microscopy (AFM) height profiles of the pristine GO and ED-GO bilayers

File Name: Supplementary Data 6
Description: Fourier transform infrared (FTIR) spectroscopy results of pristine GO and ED-GO samples

File Name: Supplementary Data 7
Description: X-ray photoelectron spectroscopy (XPS) C1s results and the deconvoluted results of pristine GO and ED-GO.

File Name: Supplementary Data 8
Description: Raman spectroscopy results of pristine GO and ED-GO samples.

File Name: Supplementary Data 9
Description: Permeances of the small gases and light hydrocarbons through ED-GO@PSSHF membrane in single gas measurement (⍰P=2 bar and room temperature)

File Name: Supplementary Data 10
Description: Separation performance (i.e. permeance and separation factor) of ED-GO@PSSHF membrane for binary gas mixtures (the volume ratio of mixture is 1:1, ⍰P=2 bar and room temperature).

File Name: Supplementary Data 11
Description: Separation performance (i.e. flux and separation factor) of ED-GO@PSSHF membrane for binary alcohol-water mixture by pervaporation at 70 o C (the

weight ratio of mixture is 1:1)

File Name: Supplementary Data 12
Description: Comparison of ED-GO@PSSHF membrane performance (i.e. flux and separation factor) for the separation of methanol-water mixture with the reported polymeric membranes, zeolite membranes, silica membranes and GO based membranes.

File Name: Supplementary Data 13
Description: Desalination performance (i.e. ion rejection and flux) of ED-GO@PSSHF membrane for different saline solutions by the vacuum membrane distillation at 60 o C.
